# Activity Identification, Classification, and Representation of Wheelchair Sport Court Tasks: A Method Proposal

**DOI:** 10.3390/mps7050084

**Published:** 2024-10-18

**Authors:** Mathieu Deves, Christophe Sauret, Ilona Alberca, Lorian Honnorat, Yoann Poulet, Arnaud Hays, Arnaud Faupin

**Affiliations:** 1 Laboratoire Jeunesse Activité Physique et Sportive—Santé (J-AP2S), Université de Toulon, 83130 La Garde, France; mdeves@fft.fr (M.D.); ilona.alberca@univ-tln.fr (I.A.); lorian.honnorat@univ-tln.fr (L.H.); vp-formation@univ-tln.fr (A.F.); 2 Fédération Française de Tennis (FFT), 75016 Paris, France; 3 Centre d’Etudes et de Recherche sur l’Appareillage des Handicapés (CERAH), Institution Nationale des Invalides, 75007 Paris, France; yoann.poulet@invalides.fr; 4 Institut de Biomécanique Humaine Georges Charpak (IBHGC), Arts et Métiers Institute of Technology, 75013 Paris, France; 5 Health Improvement Through Physical Exercice (HIPE) Human Lab, Aix Marseille Université, 13005 Marseille, France; arnaud.hays@univ-amu.fr

**Keywords:** symbolic aggregate approximation, monitoring, performance, paralympic

## Abstract

Background: Monitoring player mobility in wheelchair sports is crucial for helping coaches understand activity dynamics and optimize training programs. However, the lack of data from monitoring tools, combined with a lack of standardized processing approaches and ineffective data presentation, limits their usability outside of research teams. To address these issues, this study aimed to propose a simple and efficient algorithm for identifying locomotor tasks (static, forward/backward propulsion, pivot/tight/wide rotation) during wheelchair movements, utilizing kinematic data from standard wheelchair mobility tests. Methods: Each participant’s wheelchair was equipped with inertial measurement units—two on the wheel axes and one on the frame. A total of 36 wheelchair tennis and badminton players completed at least one of three proposed tests: the star test, the figure-of-eight test, and the forward/backward test. Locomotor tasks were identified using a five-step procedure involving data reduction, symbolic approximation, and logical pattern searching. Results: This method successfully identified 99% of locomotor tasks for the star test, 95% for the figure-of-eight test, and 100% for the forward/backward test. Conclusion: The proposed method offers a valuable tool for the simple and clear identification and representation of locomotor tasks over extended periods. Future research should focus on applying this method to wheelchair court sports matches and daily life scenarios.

## 1. Introduction

Measuring and monitoring wheelchair mobility are essential both in daily life and in sports contexts. In daily life, the activity level of manual wheelchair users serves as an important indicator of their quality of life and overall health status [1]. Several studies have focused on long-term mobility characteristics, employing tools such as accelerometers [2], data loggers [1], and machine learning algorithms capable of classifying movements [3]. In wheelchair court sports (WCSs) such as wheelchair basketball (WBas), wheelchair rugby (WRug), wheelchair tennis (WTen), or wheelchair badminton (WBad), monitoring wheelchair mobility during matches and training sessions can lead to a deeper understanding of game dynamics and of the athlete’s effort. Monitoring both external and internal loads helps to provide periodized training prescriptions and individualized training programs, while also aiding in the prevention of fatigue and injuries [4]. Numerous studies have sought to quantify the physical demands placed on WCS athletes during competition using miniaturized data loggers [5], video cameras [6], heart rate monitors [7,8], indoor wireless tracking systems [8,9], or perceived efforts collected through Borg scales [10]. These investigations have characterized WCSs as intermittent aerobic activities punctuated by brief periods of high-intensity work [11,12,13]. High-intensity actions typically involve multidirectional movements with rapid accelerations and high-speed rotations [14,15], except for WBad, which is predominantly characterized by unidirectional movements [16]. However, none of these studies have precisely described the characteristics of each locomotor task, which is essential for a deeper understanding of the sport. The use of data loggers has proven to be ineffective, as measurement errors occur at high speeds [17]. Workload tracking techniques have evolved with the advent of Global Positioning System (GPS), which allows for the quantification of the location, volume, intensity, and frequency of performed activities [18]. However, WCSs are predominantly played indoors, where GPS is unreliable, and the relatively small court dimensions require higher levels of precision and detail [17]. A radio-frequency-based indoor tracking system (ITS) has been developed, utilizing ultra-wideband signals to communicate with compact tags worn by athletes, providing real-time analysis of movement parameters [9]. However, implementing the ITS in practical terms requires substantial setup and calibration. Additionally, no data have been reported so far regarding acceleration or angular velocity using this system. Finally, in WTen, activity patterns have been studied by defining physical variables such as effective playing time or total resting time, as well as technical aspects like the type of shot or number of winning shots, using video analysis [10,19]. However, this approach requires a team of reviewers to manually document each event, which is labor-intensive and time-consuming. Therefore, these tools have proven inadequate for describing locomotor tasks due to issues of reliability, cost, or time efficiency.

Technological advancements have led to the development of smaller, lighter, and wireless inertial measurement units (IMUs). In recent years, these devices have become more accessible to research teams and sports federation staff worldwide, making field experiments feasible and yielding more ecologically valid results. Their use in WCSs is now well established and has been shown to reliably assess wheelchair kinematics [20]. IMUs are capable of collecting extensive data on linear and rotational speed, as well as acceleration performance. The three-sensor IMU configuration, which provides more robust measurements for both linear and non-linear movements [20], has been employed in numerous studies. For instance, it has been used to validate field tests for profiling purposes in WTen [21], to evaluate player performances during structured field tests [22,23], and to examine the effectiveness of different wheelchair configurations [24,25]. During WCS matches, three IMUs have also been used to identify the key characteristics of primary movements [14,15]. While many of these techniques offer satisfactory results for standard action recognition and general analysis, recognizing actions over extended periods of time—especially when dealing with large datasets from sources like video cameras—can become laborious and time-consuming in terms of data processing and interpretation. Additionally, existing IMU-based methods tend to be too generic. They often fail to provide detailed insights into the frequency and intensity of various movements, and they lack information regarding the number, duration, and distance of sprints and rotations. These data have become essential for enabling coaches and physical trainers to develop evidence-based approaches and training programs [26]. However, while large amounts of data are needed to achieve this goal, massive data collection without proper translation into actionable insights can hinder coaches. The vast range of data provided by these tools, along with the way sports scientists present them, may not seem relevant to coaches in practical training contexts unless they are simplified [27]. Amidst the growing volume of data in sports, the integration of data exploration techniques into time series analysis has led to the concept of Time Series Data Mining [28]. One notable technique is Symbolic Aggregate Approximation (SAX), which transforms time series data into symbolic form, reducing its dimensionality by converting the original data into symbolic string alphabets [29]. This method has notably been used for human action recognition [30] but has yet to be applied to wheelchair movements. Implementing this method could assist sports federations by providing a tool for identifying characteristic movements in their sport using small, non-invasive sensors, while also significantly reducing processing time compared to traditional video analysis.

This study aimed to describe and test the application of the SAX method for identifying five prevalent and distinct tasks in wheelchair court sports, including static positioning, forward propulsion, backward propulsion, pivot rotation, tight rotation, and wide rotation. We hypothesized that the algorithm would accurately detect locomotor tasks, achieving a coefficient of variation (CV) of less than 5% compared to the actual movements performed.

## 2. Methods

### 2.1. Participants

Data collection involving wheelchair badminton and tennis players was approved by the National Ethics Committee for Research in Physical Activity and Sports Sciences (CERSTAPS, IRB00012476-2021-11-06-274 and CERSTAPS n° IRB00012476-2021-31-03-97). A total of 36 wheelchair athletes participated in this study, including eighteen international wheelchair tennis players (twelve men and six women) and eighteen national wheelchair badminton players (eleven men and seven women). Among the wheelchair tennis players, thirteen competed in the Open division and five in the Quad division. The Open division, which is divided into male and female categories, consists of players with permanent lower limb impairments. In contrast, the Quad division includes athletes with permanent impairments in both upper and lower limbs, where men and women compete together [31]. Among the wheelchair badminton players, eleven competed in the Wheelchair 1 category (WH1) and seven in the Wheelchair 2 category (WH2). The WH1 category includes manual wheelchair users with abdominal and lower limb paralysis, while the WH2 category includes users with abdominal capabilities and lower limb paralysis who have partial sensation. These athletes may sometimes move in a vertical position using crutches or prostheses, but they practice the sport exclusively in a wheelchair [32]. Inclusion criteria required participants to be at a national level or higher and to have a minimum of one year of experience in the sport. Participants were excluded if they reported any pain or injuries that could hinder their ability to propel their wheelchair. Participants’ characteristics are summarized in Table 1.

### 2.2. Protocols

The data used in this article were collected during the French Wheelchair Badminton Championships and during wheelchair tennis training sessions conducted throughout the year 2023. The tests presented below were selected by the staff for their relevance in evaluating player performance relative to the characteristics of their respective discipline. These standardized and well-known tests were chosen for this study because they encompass various locomotor tasks commonly encountered in WCSs. After being informed of the protocol, participants signed a written informed consent form and completed a warm-up before performing one of the three mobility tests proposed in this study: the star test, the figure-of-eight test, or the forward/backward test. All tests were conducted as quickly as possible. The star test involved five long back-and-forth displacements with alternating 180° and 120° turns (*n* = 12). The outcome of this test was the time taken to complete the course. The figure-of-eight test required participants to navigate a course that involved crossing directions and making two turns around a spot separated by five meters, allowing for straightforward displacement between turns, for a duration of one minute (*n* = 10). The forward/backward test involved forward and backward propulsion in a straight line over a distance of three meters for one minute (*n* = 18). The outcome for these two tests was the number of complete figure-of-eight or forward/backward displacements achieved. Participants used their own wheelchair, and all tests were conducted with the racket held in their dominant hand. Four wheelchair tennis players performed both the star and figure-of-eight tests on different days.

### 2.3. Equipment

**Figure 1:**
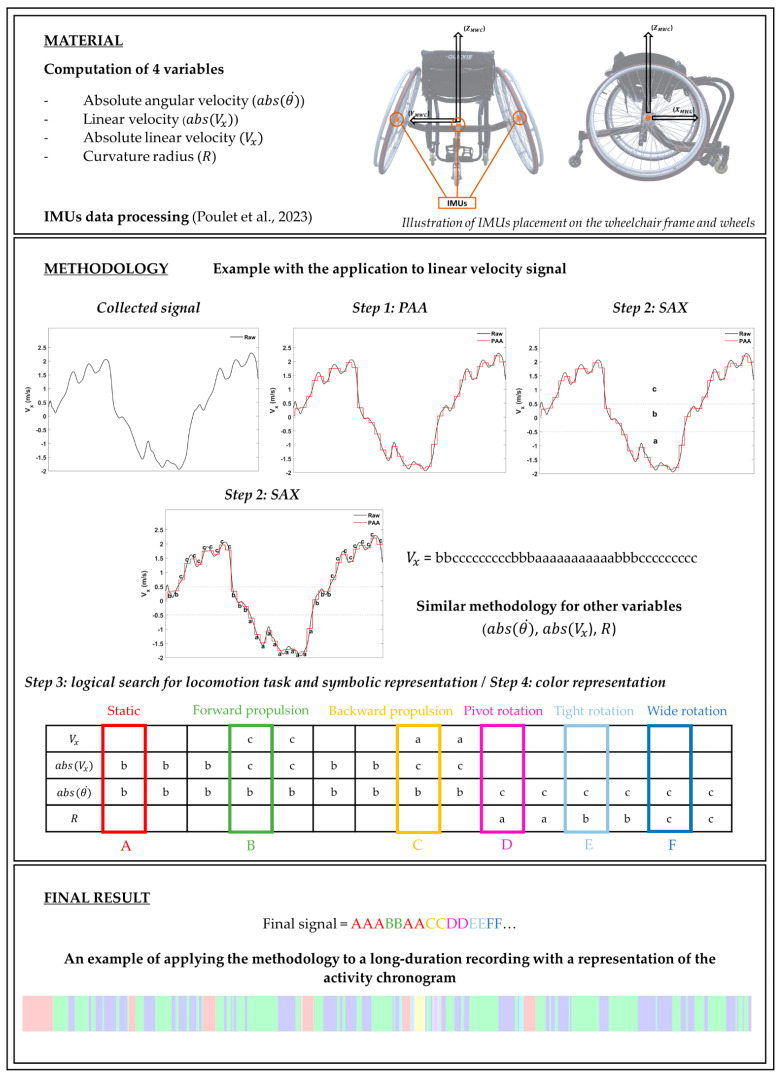
Overview of the methodology presented in this study to identify and represent the locomotor tasks performed during manual wheelchair locomotion [33].

Personal wheelchairs were equipped with three three-dimensional (*x*-, *y*-, and *z*-axis) wireless inertial measurement units (IMUs) that measured linear acceleration, angular velocity, and magnetic field orientation (WheelPerf System, AtoutNovation, Versailles, France, 128 Hz). The IMUs were positioned on the wheelchair’s frame, at the midpoint between the two rear wheel centers, and on each rear wheel hub (Figure 1). Each data acquisition was filmed using a tablet connected to the IMUs. The video recordings served as a reference to address any errors or questions related to the interpretation of the algorithm’s results.

### 2.4. Data Processing

IMU data, particularly gyroscope data, were processed using the method extensively detailed in the study by Poulet et al. [33], which is based on equations described in the works of Pansiot et al. [34] and Fuss [35]. Following this processing, four main variables were prioritized and utilized in the method presented below:

- The wheelchair linear velocity ($V_{x}$) [in m/s] (more specifically, the velocity of the midpoint between both rear wheels centers), determined from IMUs on both rear wheels (obtained under the assumption that both rear wheels are rolling without slipping on the ground);
- The absolute value of wheelchair linear velocity (abs($V_{x}$)) [in m/s];
- The absolute value of the angular velocity (abs($\dot{\theta }$)) of the wheelchair around the vertical axis [in °/s], determined from the IMU placed on the frame of the wheelchair;
- The wheelchair curvature radius (R), expressed in the MWC coordinate system and aligned with the line passing through the centers of the rear wheels, under the condition of rolling without slipping of both rear wheels [in m]. It is derived from the following equation (Equation (1)), which is based on linear and angular velocities. R represents the distance between the center of the wheelchair frame and the point around which the wheelchair rotates.
  (1) $$R=abs\left(\frac{V_{x}}{\dot{\theta }}\right)$$

### 2.5. Symbolic Time Series Analysis

The purpose of the following algorithm was to provide a symbolic representation of displacement that highlights the different locomotor tasks performed. The targeted locomotor tasks included the static phase, forward propulsion, backward propulsion, pivot rotation, tight rotation, and wide rotation. Rotations were recorded for both the right and left sides. These tasks were selected because they are the most commonly encountered in everyday activities and sports. The objective of the algorithm was to transform four synchronized time series (abs$\left(\dot{\theta }\right)$, $V_{x}$, abs$\left(V_{x}\right)$ and R) into a symbolic time series representing the locomotion tasks. To achieve this, the algorithm consists of five steps.

#### 2.5.1. Step 1: Data Reduction

After processing and highlighting the four variables mentioned above, the time series (T) of each variable was transformed into segments using Piecewise Aggregate Approximation (PAA), in which the length n of T was divided into w equal-sized “frames” [36]. In this study, a PAA segment was established every 25 values, and the mean value of the data within each segment was retained, as illustrated by the red curve in Figure 1. A vector composed of these mean values served as the data-reduced representation. The objectives of this step were to reduce the size of the signal and to decrease the variability of the identified situations.

#### 2.5.2. Step 2: Symbolic Aggregate Approximation (SAX)

After reducing T, the previously calculated mean values for each PAA segment were replaced by alphabetic values of ‘a’, ‘b’, or ‘c’, based on predetermined thresholds specified in Table 2. This transformation resulted in the four signals being converted into string vectors (SAX signals) (Figure 1). The thresholds were defined to identify the following:

- abs($V_{x}$): absence (b) or presence (c) of forward/backward motion;
- $V_{x}$: backward (a) or forward (c) motion;
- abs($\dot{\theta }$): absence (b) or presence (c) of turning motion;
- R: pivot (a), tight (b) or wide (c) rotations.

The values of these thresholds were arbitrarily adjusted based on the prior knowledge of the research team.

#### 2.5.3. Step 3: Logical Search for Locomotion Task and Symbolic Representation

From the four signals transformed into SAX signals, combinations of lowercase letters among the signals were analyzed to define the different locomotor tasks. As described in Table 3 and summarized in Figure 1, logical patterns were employed to identify these tasks, which were detected when the series of value combinations matched one of the predefined locomotor tasks. To summarize, five locomotor tasks were selected for detection and defined as follows:

- The static phase is defined by the absence of motion both in translation and rotation, which means both $V_{x}$ and $\dot{\theta }$ are equal to zero. Based on the thresholds presented in Table 2, this means that the $abs(V_{x})$ series contained ‘b’ (i.e., $abs(V_{x})$ < 0.5 m/s) and that the $abs(\dot{\theta })$ series also contained ‘b’ (i.e., $abs(\dot{\theta })$ < 40°/s) at the same index.
- The forward propulsion was defined by the presence of motion in translation, directed forward, and by the absence of rotation. Based on the thresholds presented in Table 2, this means that the $abs(V_{x})$ series contained ‘c’ (i.e., $abs(V_{x})$ > 0.5 m/s), the $V_{x}$ series contained ‘c’ (i.e., $V_{x}$ > 0.5 m/s), and the $abs(\dot{\theta })$ series contained ‘b’ (i.e., $abs(\dot{\theta })$ < 40°/s) at the same index.
- The backward propulsion was defined by the presence of motion in translation, directed backward, and by the absence of rotation. Based on the thresholds presented in Table 2, this means that the $abs(V_{x})$ series contained ‘c’ (i.e., $abs(V_{x})$ > 0.5 m/s), the $V_{x}$ series contained ‘a’ (i.e., $V_{x}$ < 0.5 m/s), and the $abs(\dot{\theta })$ series contained ‘b’ (i.e., $abs(\dot{\theta })$ < 40°/s) at the same index.
- The pivot rotation was defined by the absence of motion in translation, the presence of rotation, and by a short radius of gyration (ideally 0 m). Based on the thresholds presented in Table 2, this means that the $abs(\dot{\theta })$ series contained ‘c’ (i.e., $abs(\dot{\theta })$ > 40°/s), and that the R series contained ‘a’ (i.e., R < 0.2 m) at the same index.
- The tight rotation was defined by the low translational motion that accompanied the rotation, resulting in a medium radius of gyration. Based on the thresholds presented in Table 2, this means that the $abs(\dot{\theta })$ series contained ‘c’ (i.e., $abs(\dot{\theta })$ > 40°/s), and that the R series contained ‘b’ (i.e., 0.2< R < 0.5 m) at the same index.
- The wide rotation was defined by the presence of rotational motion largely accompanied by a translational motion, resulting in a large radius of gyration. Based on the thresholds presented in Table 2, this means that the $abs(\dot{\theta })$ series contained ‘c’ (i.e., $abs(\dot{\theta })$ > 40°/s), and that the R series contained ‘c’ (i.e., R > 0.5 m) at the same index.

Hence, when the combination of signals matched one of the predefined patterns (see Table 3), an uppercase letter representing the detected locomotor task was assigned to a new field. The letters were defined as follows: ‘A’ for the static phase, ‘B’ for forward straight-line movement, ‘C’ for backward straight-line movement, ‘D’ for pivot rotation, ‘E’ for tight rotation, and ‘F’ for wide rotation. Consequently, the final resulting signal was composed solely of these letters, which represented the wheelchair user’s activity (Figure 1).

#### 2.5.4. Step 4: Color Representation

To provide a clearer representation of locomotor tasks, each letter was associated with a specific color for graphical visualization. As detailed in Table 3 and illustrated in Figure 1, the use of colors facilitates a more visual and simplified description of the locomotor tasks performed over extended periods of time. The colors assigned were as follows: red for ‘A’ (static phase), green for ‘B’ (forward propulsion), yellow for ‘C’ (backward propulsion), pink for ‘D’ (pivot rotation), cyan for ‘E’ (tight rotation), and blue for ‘F’ (wide rotation).

**Table 3:** Identification of locomotor tasks based on signal combination (step 3) and assignment of a letter and color to each locomotor task (step 4).

	Static	Forward Propulsion	Backward Propulsion	Pivot Rotation	Tight Rotation	Wide Rotation
$V_{x}$		c	a			
$abs(V_{x})$	b	c	c			
$abs(\dot{\theta })$	b	b	b	c	c	c
R				a	b	c
	A	B	C	D	E	F

#### 2.5.5. Step 5: Reduction in Variability

Finally, after all locomotor tasks were detected, a last processing step was implemented to reduce the variability of actions. This involved instructing the algorithm that if a segment of one locomotor task was surrounded by multiple segments of another locomotor task, it would adopt the designation of the predominant locomotor task, thereby creating a unified action. For instance, if the algorithm detected several consecutive segments of straight-line movement, but a single segment of wide turn appeared in between, that wide turn would be transformed into a straight-line segment.

### 2.6. Data Analysis

Since the chosen tests were standardized with precise and well-defined trajectories, the locomotor tasks for forward propulsion, backward propulsion, and rotations were quantified using the algorithm. These counts were then compared with the number of round trips recorded by the operator during the forward/backward test and the number of laps counted during the figure-of-eight test on the day of experimentation. Due to the visual indistinguishability of pivot rotation, tight rotation, and wide rotation during the experiment, these locomotor tasks were grouped together in the analysis. The algorithm also provided additional insights into the athletes’ movement strategies throughout the tests. To assess the accuracy of the movements recorded by the operator versus the locomotor tasks detected by the algorithm, the coefficient of variation (CV) was employed. For each subject, the comparison between the two analysis methods was expressed as a percentage, allowing for a detailed evaluation of the algorithm’s performance in detecting locomotor tasks.

## 3. Results

**Figure 2:**
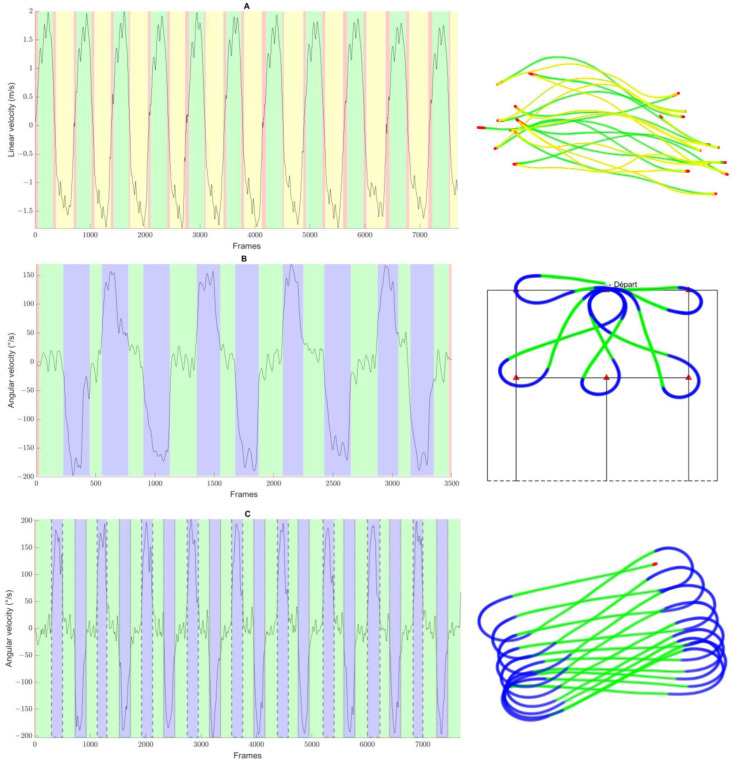
Identification of locomotor tasks and representation of trajectories (on the right) from the three field tests conducted ((**A**) forward/backward test; (**B**) star test; (**C**) figure-of-eight-test). Each color is associated with locomotor tasks (green: forward propulsion; yellow: backward propulsion; red: static phase; blue: wide rotation). On the left side, colors are overlaid on the yaw angular velocity signals of the wheelchair. In the graph representing the locomotor tasks of the figure-of-eight test, the frame composed of dashed lines is used to represent left turns (positive values), and the frame composed of dots is used to represent right turns (negative values).

**Figure 3:**
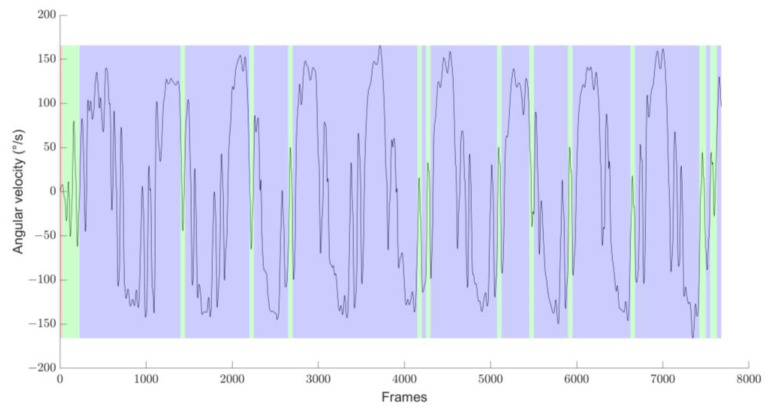
Representation of the locomotor tasks detected using the SAX algorithm for the athlete with asynchronous propulsion during the figure-of-eight test. The colors are overlaid on the yaw angular velocity signals of the wheelchair that exhibit alternance of positive and negative values, meaning that the turning direction changed during the full recognized wide rotation task.

Table 4 summarizes the number of locomotor tasks inferred during the experimentations and detected by the algorithm for each test. The results demonstrate that the proposed algorithm accurately identified locomotor tasks, as indicated by the low coefficient of variation (maximum of 3.6% for the figure-of-eight test). These findings are further supported by the graphical representation of locomotor activity and wheelchair trajectory across the three tests conducted (Figure 2). However, in one instance of the figure-of-eight test, 20 straight lines and 19 rotations were performed, while the algorithm only detected 12 straight lines and 12 rotations (Figure 3). This represents a reduction of 40% in the locomotor tasks detected by the algorithm.

## 4. Discussion

The objective of this study was to propose a simple and effective method for detecting and representing locomotor tasks using gyroscope data from inertial measurement units. We hypothesized that the proposed method would accurately identify all targeted locomotor tasks: static; forward and backward propulsion; and pivot, tight, and wide rotations. The evaluation presented in this article demonstrates that the use of IMU gyroscope data, coupled with the proposed algorithm, allows for accurate identification of the locomotor tasks performed. This method has been notably applied to a battery of tests conducted at high intensity by national-level wheelchair tennis and wheelchair badminton athletes. Based on the proposed thresholds, all recordings were classified into locomotor tasks as instructed by the algorithm. For each test, we compared the number of locomotor tasks inferred from the total number of round trips or laps completed with the number of tasks identified by the algorithm, thereby validating the method employed. This comparison revealed an average similarity of 99% for the star test, 100% for the forward/backward test, and 95% for the figure-of-eight test. Additionally, the visual representation of locomotor tasks on the course trajectory (Figure 2) further reinforces the validity of the employed method.

The analysis technique used in this study to measure wheelchair movement, along with the performed validation, presents several advantages and limitations compared to existing methods for monitoring wheelchair activity. The proposed algorithm can complement the overall analysis conducted during matches by utilizing the IMUs installed on the wheelchair [14,15], providing a detailed and representative description of each movement performed. Information such as the number of sprints, their duration, the distance covered, and the types of rotations are essential elements for coaches to understand athlete performance. Other studies have also characterized on-field mobility intensity during matches using arbitrary speed zones [5,37]. However, these processing methods do not provide a precise description of locomotor tasks or their evolution throughout the match. The proposed methodology could be employed to better understand mobility characteristics during a match and to analyze movement strategies based on factors such as score progression, fatigue, and other influences. Indeed, characterizing the intensity of locomotor tasks represents a significant area for further research. For each locomotor task, it would be possible to analyze the movement performed from the start to the end of recognition using key kinematic performance metrics extracted from IMUs. These metrics could include maximum linear velocity, average linear velocity, duration, and distance for straight-line propulsion, as well as rotation angle, maximum angular velocity, and curvature radius evolution for rotations.

Another advantage of the proposed methodology is its versatility, making it applicable in various contexts, including everyday life and sports, provided that the thresholds used are validated. Additional signals derived from the same initial IMU data can be incorporated, and the algorithm’s thresholds can be adjusted to suit different applications, such as monitoring mobility in daily activities or tracking movement during competitive sports like wheelchair rugby or wheelchair basketball. Future research employing this method for detecting locomotor tasks is encouraged to include detailed supplementary material that outlines segment length (PAA step), signals used, applied thresholds (SAX step), and the pattern recognition methods utilized for task identification.

In the present study, the algorithm detected 40% fewer locomotor tasks for one out of the ten recordings of the figure-of-eight test. This discrepancy can be attributed to the different propulsion strategies employed by the athletes. Specifically, this athlete used asynchronous propulsion—alternating propulsion between the left and right wheels—which resulted in frame rotation around its vertical axis. Consequently, while a straight-line locomotor task was expected during the test, the algorithm identified wide rotations instead. A more detailed analysis based on the video data reveals that the actual trajectory of the wheelchair athlete was less straightforward than anticipated. In wheelchair sports, some athletes favor asynchronous propulsion, and coaches may prefer that certain propulsion segments performed in this mode be classified as straightforward rather than as alternating left/right wide rotations. To address this, additional correction steps could be implemented, such as including the direction of angular velocity as an input signal and establishing a threshold for maximum global rotation per push.

Despite these limitations, this article demonstrates the feasibility of analyzing mobility characteristics using gyroscopic data and the proposed algorithm. While the thresholds were manually tuned based on team experience—an aspect that may be considered a limitation—the proposed thresholds have proven effective in the context of standardized tests. Consequently, both the signals and thresholds presented in this study provide a foundational framework that can be adjusted for greater specificity in the algorithm’s application to different types of wheelchair use or for more refined task detection. Moreover, this method offers a simple and clear representation for coaching staff during activity analysis. Finally, future research applying this processing method to wheelchair sports matches could enhance our understanding of mobility characteristics. Additionally, extending this methodology to monitor locomotor tasks in daily life could complement existing approaches.

## Figures and Tables

**Table 1:** Participants’ characteristics, mean (SD).

		Wheelchair Tennis		Wheelchair Badminton	
Characteristics	Total (*n* = 36)	Open (*m* = 8/*f* = 5)	Quad (*m* = 4/*f* = 1)	WH1 (*m* = 7/*f* = 4)	WH2 (*m* = 4/*f* = 3)
Age (years)	40 (9.5)	36.2 (11.1)	44 (5.8)	43.9 (6.1)	40 (10.9)
Mass (kg)	66.7 (13)	66.7 (16.9)	69.4 (11.5)	69 (10)	61 (9.9)
Years of training	9.2 (6.5)	12.3 (8)	Unknown	7 (2.7)	7.1 (5.3)

**Table 2:** A summary of the thresholds used to replace the PAA segment values into classes labeled a, b, or c.

	a	b	c
$V_{x}$ **(m/s)**	≤−0.5	−0.5 < v < 0.5	≥0.5
$abs(V_{x})$ **(m/s)**		<0.5	≥0.5
$abs(\dot{\theta })$ **(°/s)**		<40	>40
R **(m)**	≤0.2	0.2 < R < 0.5	≥0.5

**Table 4:** A summary of the number of locomotor tasks counted both through observations and detected by the SAX method across the three tests, mean (SD).

Tests	Figure-of-Eight Test		Star Test		FP-BP Test	
	FP	Rotations	FP	Rotations	FP	BP
Observations	20 (1.3)	19 (1.6)	10	9	12 (1.8)	11 (1.9)
SAX method	19 (2.7)	19 (3.0)	10 (0.6)	9 (0.3)	12 (1.8)	11 (1.9)
CV (%)	3.6	3.4	1.2	0.7	0	0

*Note*. FP: forward propulsion; BP: backward propulsion; CV: coefficient of variation.

## Data Availability

Data are available upon reasonable request from the corresponding author.
